# Development of a Novel Prognostic Inflammation Index to Predict Poor Outcomes in Patients With Intracerebral Hemorrhage: A Longitudinal Study

**DOI:** 10.1002/cns.70904

**Published:** 2026-04-27

**Authors:** Guangyong Chen, Feng Chen, Xin Lu, Zhangjing Zhu, Fangyan Chen, Qian Liu, Ziming Ren, Changhao Zhang, Yuxin Zhu, Yueping Chen, Suwen Huang, Dehao Yang, Yiyun Weng

**Affiliations:** ^1^ Department of Neurology The Third Affiliated Hospital of Wenzhou Medical University Wenzhou Zhejiang China; ^2^ Department of Neurology The First Affiliated Hospital of Wenzhou Medical University Wenzhou Zhejiang China; ^3^ The Second School of Medicine Wenzhou Medical University Wenzhou Zhejiang China; ^4^ The First School of Medicine, School of Information and Engineering Wenzhou Medical University Wenzhou China; ^5^ The School of Mental Health Wenzhou Medical University Wenzhou Zhejiang China; ^6^ The School of Rehabilitation Wenzhou Medical University Wenzhou Zhejiang China; ^7^ Clinical Laboratory The Third Affiliated Hospital of Wenzhou Medical University Wenzhou Zhejiang China; ^8^ Department of Neurology The Second Affiliated Hospital, Zhejiang University School of Medicine Hangzhou Zhejiang China

**Keywords:** inflammation, intracerebral hemorrhage, leukocyte subsets, prognosis, prognostic inflammation index

## Abstract

**Background:**

Spontaneous intracerebral hemorrhage (ICH) is an acute cerebrovascular disease associated with high mortality and severe disability. Inflammation plays a critical role in the onset and progression of ICH. However, existing inflammatory markers have limited predictive capacity for the prognosis of ICH patients. This study aims to develop a novel Prognostic inflammation index (PII) based on leukocyte subset counts and evaluate its effectiveness in assessing the prognosis of ICH patients.

**Methods:**

A total of 1021 consecutive ICH patients hospitalized between January 2021 and June 2023 were included as the derivation cohort. In addition, an internal temporal validation cohort of 366 patients hospitalized between January 2024 and December 2024 was assembled using identical inclusion/exclusion criteria. Using reduced‐rank regression (RRR) based on leukocyte subsets (including neutrophils, monocytes, lymphocytes, eosinophils, and basophils), we constructed the PII. Multivariate logistic regression was employed to analyze the associations between PII, its dynamic trajectories, and patient outcomes, including poor prognosis, all‐cause mortality, and stroke‐associated infections. The predictive performance of PII was illustrated using a nomogram, and its efficacy was compared to conventional inflammatory markers through receiver operating characteristic (ROC) curve analysis.

**Results:**

Patients with elevated PII were significantly associated with poor outcomes at 3, 6, and 12 months, stroke‐associated infections, and all‐cause mortality within 1 year (all *p* < 0.05). PII trajectory analysis revealed that patients with persistently high PII had a substantially increased risk of poor outcomes (*p* < 0.05). Moreover, compared to common systemic inflammatory markers such as the systemic immune‐inflammation index (SII), systemic inflammation response index (SIRI), and aggregate inflammation systemic index (AISI), PII showed generally favorable discriminative performance across most endpoints; however, the pairwise AUC comparisons were exploratory and some comparisons yielded borderline *p* values that should be interpreted cautiously.

**Conclusion:**

PII, a composite inflammation index based on leukocyte subset counts, is an effective predictor of poor outcomes in ICH patients and shows favorable prognostic performance compared with traditional inflammatory markers.

## Graphical abstract

We developed a novel Prognostic Inflammation Index (PII) based on five circulating leukocyte subtypes in patients with primary intracerebral hemorrhage. PII independently predicted poor functional outcome, stroke‐associated infection, and 1‐year all‐cause mortality, indicating its potential value for early risk stratification and prognosis assessment.

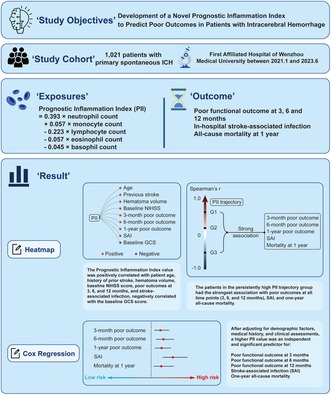

## 1 Introduction

Spontaneous intracerebral hemorrhage (ICH) accounts for 20% to 30% of all stroke types [1]. Although the incidence of ICH is lower than that of ischemic stroke, ICH imposes a greater social and economic burden and is a leading cause of global mortality and long‐term disability, particularly in low‐ and middle‐income countries [2]. ICH remains highly lethal, with an estimated 40%–50% 30‐day mortality, highlighting the urgent need to improve strategies for supportive care and prognostic management [3]. However, effective treatment options to improve the prognosis of ICH patients remain lacking [4]. Timely intervention and early outcome prediction have the potential to reduce mortality and improve outcomes in ICH patients. Therefore, identifying biomarkers capable of predicting the prognosis of ICH patients is of critical importance.

The mechanisms of inflammation in ICH are complex [5]. During brain injury following ICH, inflammation serves as a key indicator of the body's defense response. Upon ICH onset, blood enters the brain parenchyma, triggering the accumulation and activation of inflammatory cells. This process begins with the early inflammatory response of glial cells, followed by the infiltration of various circulating inflammatory cells, including the activation of neutrophils, leukocytes, and macrophages [6, 7]. Simultaneously, the inflammatory immune response mediated by the sympathetic nervous system and the hypothalamic–pituitary–adrenal (HPA) axis is activated, leaving ICH patients in a state of sustained inflammation, reducing systemic immune activity, and suppressing cellular immune responses [8, 9]. As a result, inflammation‐related biomarkers in ICH patients, as a non‐invasive and cost‐effective detection method, can further aid in predicting patient outcomes.

Leukocyte and leukocyte subset counts are key components reflecting the inflammatory state. The neutrophil‐to‐lymphocyte ratio (NLR) and white blood cell (WBC) count have been shown to be associated with poor outcomes and higher mortality in ICH patients [10]. NLR has also been demonstrated to be a reliable predictor of stroke‐associated pneumonia and poor discharge outcomes in ICH patients [11]. However, most inflammatory markers are calculated based on partial leukocyte subset counts and lack comprehensive predictive capability. Therefore, we developed a novel Prognostic inflammation index (PII) based on comprehensive leukocyte subset counts to predict poor outcomes in ICH patients and compared its predictive performance with other common systemic inflammatory markers. This provides an effective biological screening tool for predicting adverse outcomes in ICH patients.

## 2 Methods

### 2.1 Study Participants

This study included 1202 patients with primary spontaneous ICH who were hospitalized at the First Affiliated Hospital of Wenzhou Medical University between January 2021 and June 2023. The diagnosis of ICH was confirmed by cranial computed tomography (CT) at admission. Patients with traumatic ICH, subarachnoid hemorrhage, subdural hematoma, epidural hematoma, or hemorrhagic transformation of ischemic stroke were excluded. Inclusion criteria were as follows: (1) age ≥ 18 years with complete age data; and (2) availability of complete clinical data and follow‐up outcomes. Exclusion criteria were defined as [12]: (1) presence of autoimmune diseases; (2) malignancies; (3) severe liver or renal failure; and (4) infections occurring within 48 h of admission. After applying these criteria, a total of 1021 patients were included in the final analysis. All included patients received conservative medical treatment only, without surgical intervention. All analyses were conducted using a complete case approach for the variables required by each model. The extent and patterns of missing data are summarized for both the derivation and validation cohorts in Table S6, including missing 24‐h leukocyte subset counts required for PII construction, hematoma volume, baseline NIHSS or GCS assessments, covariates, and follow‐up mRS assessments. Because leukocyte subset counts are the primary predictors used to construct the PII, we did not impute these values to avoid introducing additional model dependent uncertainty. Analyses were therefore restricted to participants with complete information for the variables included in each specific analysis (Table S6). The detailed inclusion and exclusion flowchart is provided in Figure S1. To evaluate temporal robustness, we further constructed an internal validation cohort comprising 366 consecutive patients with primary spontaneous ICH admitted to the same hospital between January 2024 and December 2024. The validation cohort was entirely non‐overlapping with the derivation cohort and used the same inclusion/exclusion criteria, variable definitions, and outcome ascertainment.

This study was approved by the Ethics Committee of the First Affiliated Hospital of Wenzhou Medical University (Project No. KY2023‐R123) and was conducted in accordance with the Declaration of Helsinki. Given the retrospective design of the study, the requirement for informed consent for the analysis of existing data was waived by the Ethics Committee in accordance with national legislation and institutional requirements.

### 2.2 Data Collection and Index Construction

Demographic data (age, sex) and medical history (smoking, drinking, hypertension, diabetes, and previous stroke) were obtained from medical records. Serum biomarkers included neutrophil, monocyte, lymphocyte, eosinophil, and basophil counts measured at admission, 24 h post‐admission, and seven days post‐admission (within days 5–9 post‐admission). Stroke severity was assessed using the National Institutes of Health Stroke Scale (NIHSS) at admission and 24 h post‐admission. Baseline Glasgow Coma Scale (GCS) scores were also recorded [13]. Patient outcomes were evaluated using the modified Rankin Scale (mRS) at 3 months, 6 months, and 1 year after ICH onset. Baseline hematoma volume was calculated using the ABC/2 formula [14]. Stroke‐associated infections (SAI) were defined as any infection diagnosed during hospitalization in ICH patients [15].

In the derivation cohort, the PII was developed using reduced‐rank regression (RRR), a multivariate dimension‐reduction approach that identifies linear combinations of predictors that best explain variation in multiple response variables [16]. Leukocyte subset counts (neutrophils, monocytes, lymphocytes, eosinophils, and basophils) were treated as predictor variables, and short‐term functional outcomes (discharge mRS and 3‐month mRS, encoded on the original 0–6 scale) were treated as response variables. All leukocyte subset counts were standardized prior to RRR. RRR derived two latent factors based on leukocyte subset counts; the first factor explained the greatest proportion of variation in the response variables, whereas the second factor explained a negligible proportion (Figure S4A). Therefore, the score of the first latent factor was selected to construct the PII. The coefficients in the PII formula correspond to the RRR factor loadings for this first latent factor and reflect the relative contribution of each standardized leukocyte subset (Figure S4B):

$$\begin{array}{c} \mathrm{PII}=0.393\times \text{neutrophil count}+0.057\times \text{monocyte count}\\ -0.223\times \text{lymphocyte count}-0.057\times \text{eosinophil count}\\ -0.045\times \text{basophil count}. \end{array}$$

We further evaluated the stability of the RRR‐derived loadings in the derivation cohort using 1000 bootstrap resamples with sign alignment; the bootstrap 95% confidence intervals and sign consistency are provided in Table S7.

All leukocyte subset counts were standardized before analysis. In the internal validation cohort, leukocyte subset variables were standardized using the same procedure, and PII was calculated by directly applying the fixed RRR‐derived loadings from the derivation cohort without re‐estimating any coefficients.

### 2.3 Clinical Outcomes

The primary outcome was poor functional outcome, assessed using mRS scores (3–6) at 3 months, 6 months, and 1 year after ICH onset. Secondary outcomes included 1‐year all‐cause mortality and SAI. The mRS scores range from 0 to 6, with 0 indicating no disability, and higher scores indicating greater disability. Functional outcomes were categorized as favorable (mRS 0–2) or poor (mRS 3–6) [17].

### 2.4 Statistical Analysis

The Kolmogorov–Smirnov test was used to assess the normality of data distribution. Continuous variables were expressed as mean ± standard deviation or median (interquartile range), and categorical variables were expressed as percentages. Differences between groups were compared using Student's *t*‐tests, non‐parametric tests, or chi‐square tests, as appropriate. Patients were classified into three groups representing different inflammatory states using a group‐based trajectory model (GBTM).

Multivariate logistic regression was used to explore associations between PII, PII trajectories, and poor outcomes at 3 months, 6 months, and 1 year, as well as SAI. To assess potential multicollinearity among independent variables, we calculated the variance inflation factor (VIF). A VIF > 5 was considered indicative of significant multicollinearity. All variables included in the final regression models had VIF values < 5, suggesting no significant collinearity. Cox regression analysis was conducted to investigate the relationship between PII, PII trajectories, and 1‐year all‐cause mortality. Generalized linear mixed models were used to assess longitudinal changes in PII and its components in predicting ICH patient outcomes. Additionally, restricted cubic splines were employed to visualize the relationship between PII and poor outcomes or SAI at 3, 6, and 12 months. A nomogram was used to demonstrate the predictive efficacy of PII for patient outcomes.

## 3 Results

### 3.1 Baseline Characteristics of Patients

The baseline characteristics of patients with different prognostic levels are presented in Table 1. Based on the 3‐month mRS scores, 666 ICH patients had favorable outcomes (mRS: 0–2), while 355 had poor outcomes (mRS: 3–6). Demographically, patients with poor outcomes were older (*p* < 0.001). Clinically, these patients had larger hematoma volumes, higher baseline NIHSS scores, and lower baseline GCS scores (all *p* < 0.001). Regarding medical history, patients with poor outcomes had higher rates of hypertension and prior stroke (*p* = 0.020, *p* < 0.001) and were more likely to develop SAI during hospitalization (*p* < 0.001). Laboratory findings revealed that poor outcome patients had lower eosinophil counts, higher neutrophil and monocyte counts, lower lymphocyte and basophil counts, and higher PII values (all *p* < 0.001) (Table 1).

**TABLE 1:** Baseline characteristics of patients according to mRS levels.

Characteristics	Total (*n* = 1021)	mRS 0–2 (*n* = 666)	mRS 3–6 (*n* = 355)	*p*
Demographics				
Age (years)	63.00 (53.00, 72.00)	60.00 (53.00, 69.00)	68.00 (56.00, 75.00)	< 0.001
Sex (Male, *n*%)	686 (67.19)	456 (68.47)	230 (64.79)	0.233
Clinical status				
Hematoma volume (mL)	7.47 (3.15, 15.40)	6.06 (2.36, 11.40)	11.51 (5.92, 21.03)	< 0.001
Baseline NIHSS	5.00 (2.00, 10.00)	3.00 (1.00, 6.00)	11.00 (8.00, 14.00)	< 0.001
Baseline GCS	15.00 (13.00, 15.00)	15.00 (15.00, 15.00)	13.00 (10.00, 15.00)	< 0.001
Medical history				
Smoking, *n* (%)	355 (34.77)	236 (35.44)	119 (33.52)	0.541
Drinking, *n* (%)	384 (37.61)	261 (39.19)	123 (34.65)	0.154
Hypertension, *n* (%)	764 (74.83)	483 (72.52)	281 (79.15)	0.020
Diabetes, *n* (%)	169 (16.55)	100 (15.02)	69 (19.44)	0.070
Previous stroke, *n* (%)	186 (18.22)	95 (14.26)	91 (25.63)	< 0.001
Complication				
SAI, *n* (%)	129 (12.63)	53 (7.96)	76 (21.41)	< 0.001
Laboratory examinations				
Eosinophil count (10^9^/L)	0.05 (0.01, 0.11)	0.06 (0.02, 0.12)	0.03 (0.01, 0.08)	< 0.001
Neutrophil count (10^9^/L)	5.49 (4.20, 7.68)	4.98 (3.91, 6.66)	7.03 (5.19, 8.98)	< 0.001
Lymphocyte count (10^9^/L)	1.28 (0.96, 1.65)	1.40 (1.04, 1.73)	1.10 (0.85, 1.42)	< 0.001
Monocyte count (10^9^/L)	0.45 (0.35, 0.58)	0.43 (0.34, 0.55)	0.49 (0.37, 0.66)	< 0.001
Basophil count (10^9^/L)	0.02 (0.01, 0.03)	0.02 (0.01, 0.03)	0.02 (0.01, 0.02)	< 0.001
PII	−0.07 (−0.36, 0.31)	−0.20 (−0.46, 0.01)	0.23 (−0.10, 0.59)	< 0.001

Abbreviations: GCS, Glasgow Coma Scale; mRS, modified Rankin Scale; NIHSS, National Institute of Health Stroke Scale; PII, prognostic inflammation index; SAI, stroke‐associated infection.

### 3.2 PII Trajectory Construction and Correlation Analysis

**FIGURE 1:**
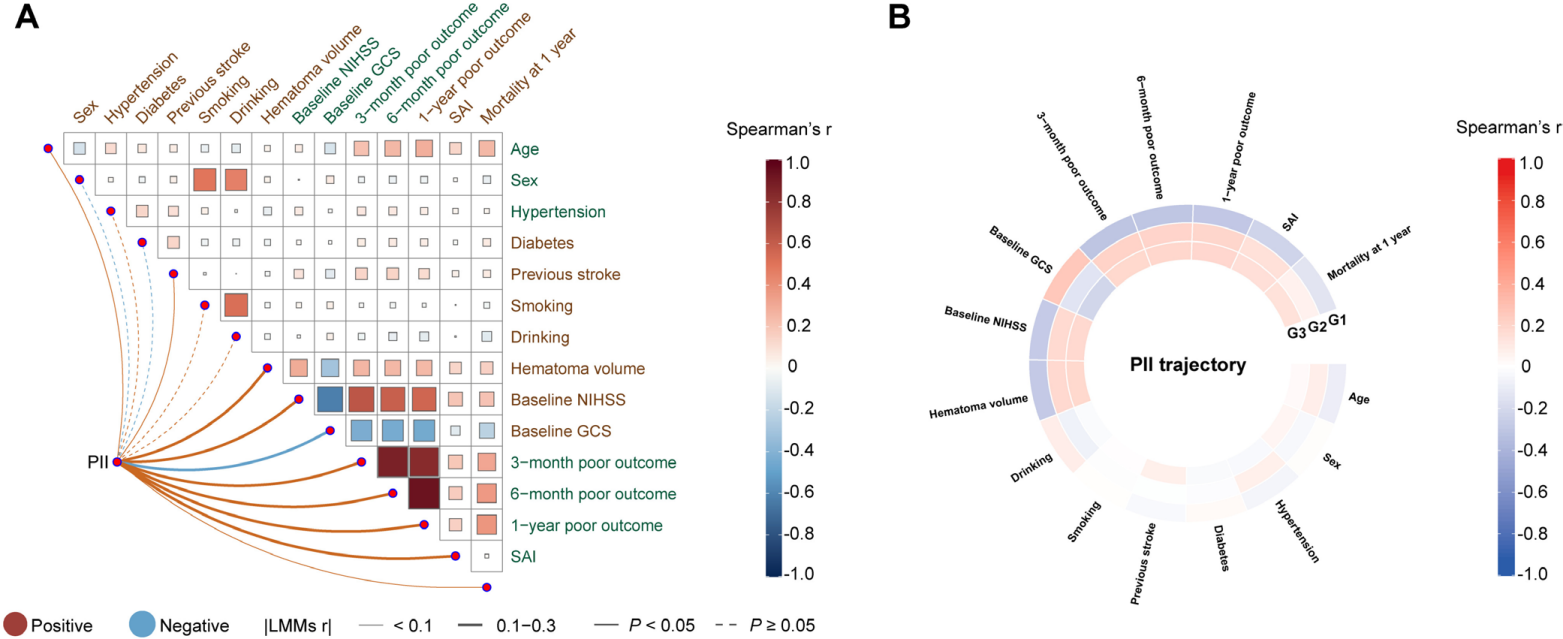
Correlation between PII and PII trajectory with various variables. (A) The correlation between PII and various variables. (B) The correlation between PII trajectory and various variables. GCS, Glasgow Coma Scale; NIHSS, National Institutes of Health Stroke Scale; PII, prognostic inflammation index; SAI, stroke‐associated infection.

To investigate the impact of inflammatory trajectories on patient outcomes, dynamic PII values at admission, 24 h, and 7 days (within days 5–9 post‐admission) were analyzed using a group‐based trajectory model, which identified three distinct PII trajectories: (1) persistently low PII, (2) moderate PII, and (3) persistently high PII (Figure S2). These trajectories represent three different inflammatory states. A heatmap analysis showed that PII was positively correlated with patient age, prior stroke, hematoma volume, baseline NIHSS, and poor outcomes at 3, 6, and 12 months, as well as with SAI, while negatively correlated with baseline GCS (Figure 1A). High PII (Group 3) was closely associated with poor outcomes and related endpoints (Figure 1B).

### 3.3 Associations Between PII, PII Trajectories, and Patient Outcomes

**FIGURE 2:**
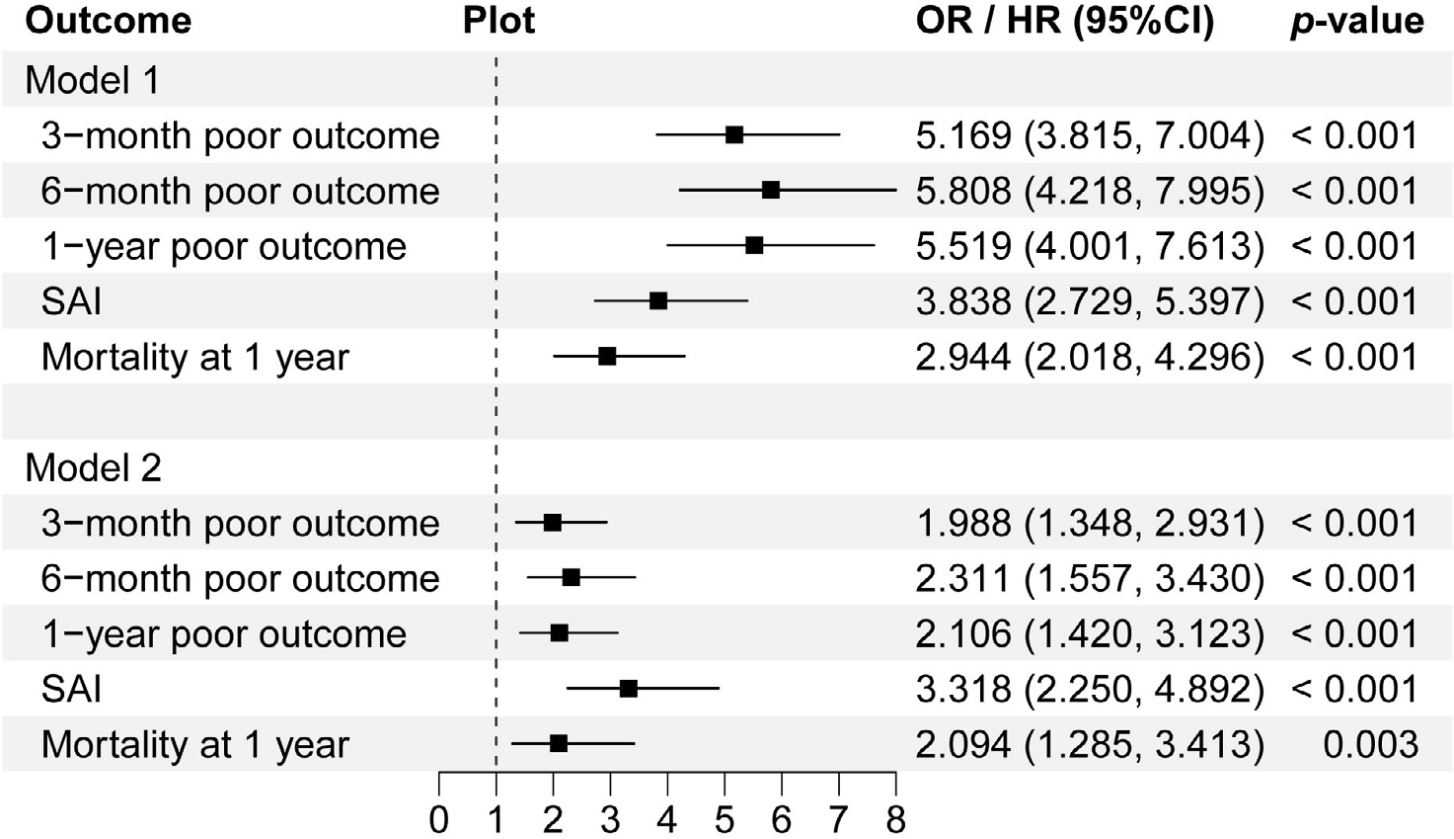
Multivariable logistic regression analysis of PII and poor outcomes at 3, 6, and 12 months, SAI, and 1‐year all‐cause mortality in ICH patients. CI, confidence interval; HR, hazard ratio; ICH, intracerebral hemorrhage; OR, odds ratio; PII, prognostic inflammation index; SAI, stroke‐associated infection.

Multivariate logistic regression was performed to explore associations between PII, PII trajectories, and poor outcomes at 3, 6, and 12 months, as well as SAI. Cox regression was used to assess the relationship between PII, PII trajectories, and 1‐year all‐cause mortality. After adjusting for demographic factors, medical history, and clinical assessments, higher PII was significantly associated with 3‐month poor outcomes [1.988 (1.348, 2.931)], 6‐month poor outcomes [2.311 (1.557, 3.430)], 1‐year poor outcomes [2.106 (1.420, 3.123)], SAI [3.318 (2.250, 4.892)], and 1‐year all‐cause mortality [2.094 (1.285, 3.413)] (Figure 2).

**FIGURE 3:**
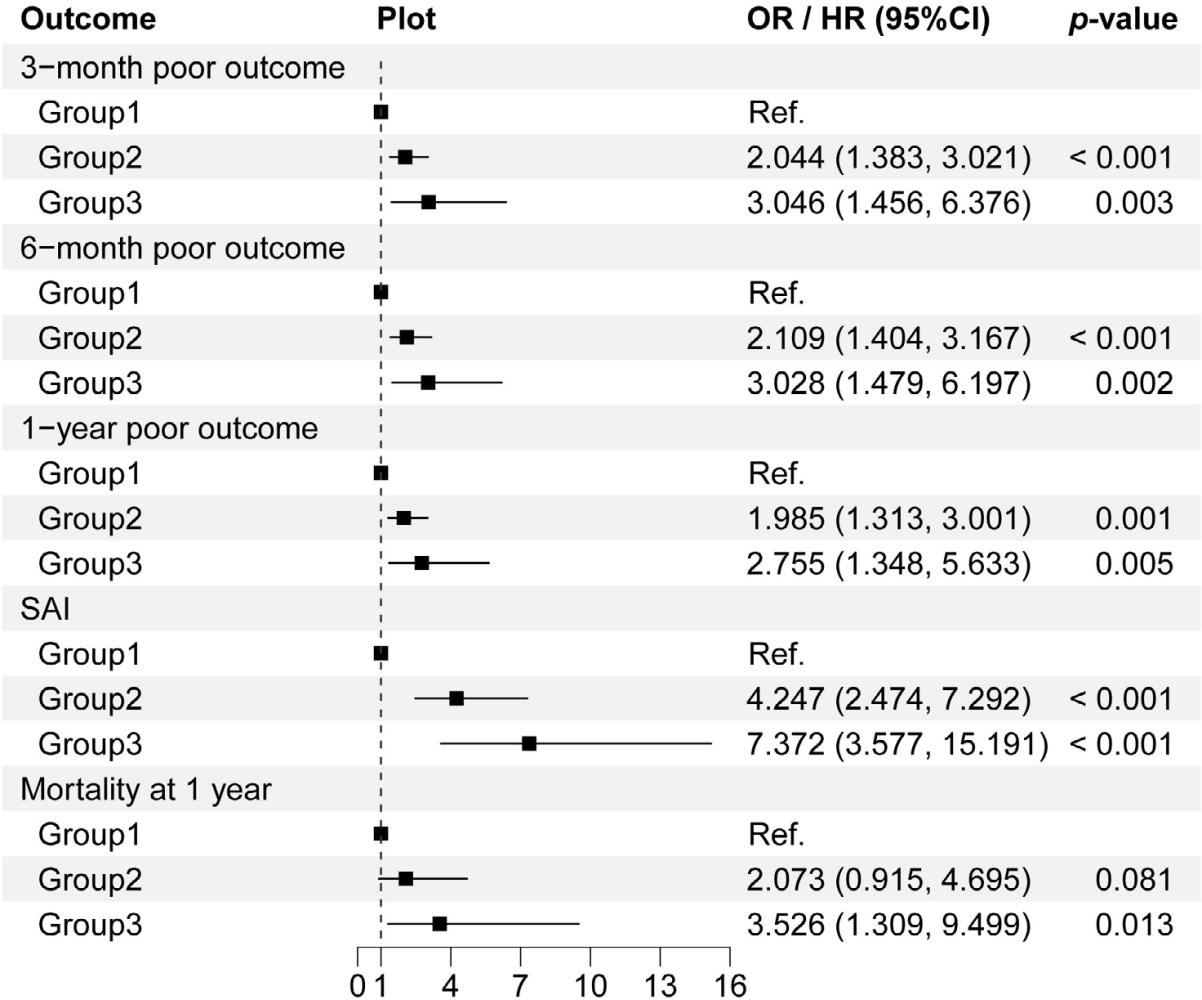
Multivariable logistic regression analysis of PII trajectory and poor outcomes at 3, 6, and 12 months, SAI, and one‐year all‐cause mortality in ICH patients. CI, confidence interval; HR, hazard ratio; ICH, intracerebral hemorrhage; OR, odds ratio; PII, prognostic inflammation index; SAI, stroke‐associated infection.

Using the low PII trajectory group (Group 1) as the reference, patients in the moderate PII trajectory group (Group 2) had significantly higher risks of 3‐month poor outcomes [2.044 (1.383, 3.021)], 6‐month poor outcomes [2.109 (1.404, 3.167)], 1‐year poor outcomes [1.985 (1.313, 3.001)], and SAI [4.247 (2.474, 7.292)]. Similarly, patients in the high PII trajectory group (Group 3) had even higher risks of 3‐month poor outcomes [3.046 (1.456, 6.376)], 6‐month poor outcomes [3.028 (1.479, 6.197)], 1‐year poor outcomes [2.755 (1.348, 5.633)], SAI [7.372 (3.577, 15.191)], and 1‐year all‐cause mortality [3.526 (1.309, 9.499)] (Figure 3).

**FIGURE 4:**
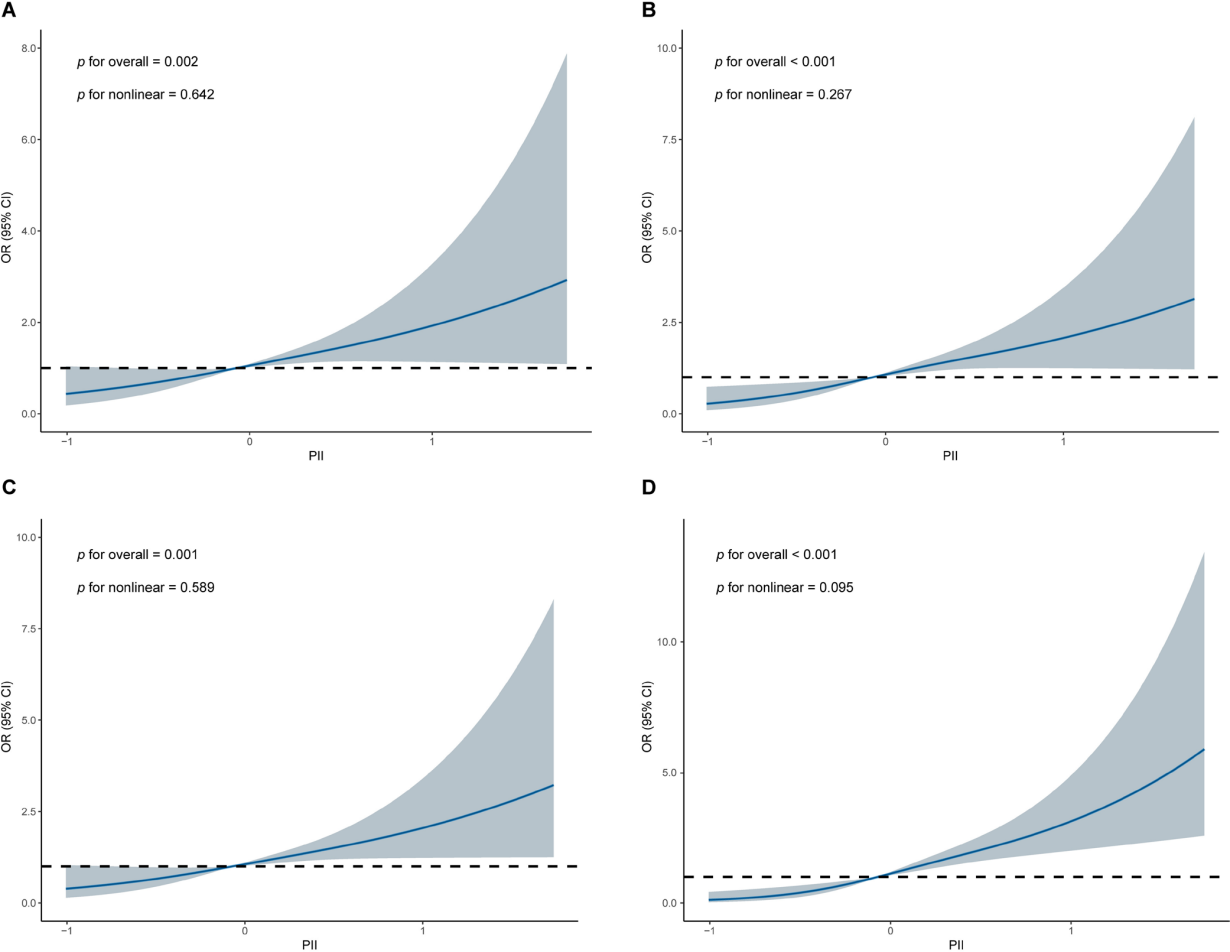
Restricted cubic spline illustrating the association between PII and poor outcomes at 3, 6, and 12 months, and SAI in ICH patients. (A) The relationship between PII and 3‐month poor outcomes in patients. (B) The relationship between PII and 6‐month poor outcomes in patients. (C) The relationship between PII and 1‐year poor outcomes in patients. (D) The relationship between PII and SAI in patients. CI, confidence interval; ICH, intracerebral hemorrhage; OR, odds ratio; PII, prognostic inflammation index; SAI, stroke‐associated infection.

Restricted cubic splines illustrated the linear relationship between PII and various outcomes, including poor outcomes at 3, 6, and 12 months and SAI (all *p* < 0.05) (Figure 4).

### 3.4 Temporal Internal Validation

In the internal validation cohort (January 2024 to December 2024; *n* = 366), higher PII remained significantly associated with poor functional outcome at 3 months (adjusted OR 2.285, 95% CI 1.171–4.458; *p* = 0.015), 6 months (adjusted OR 3.175, 95% CI 1.522–6.622; *p* = 0.002), and 1 year (adjusted OR 2.897, 95% CI 1.410–5.952; *p* = 0.004), and with stroke‐associated infection (adjusted OR 3.342, 95% CI 1.787–6.248; *p* < 0.001). For 1‐year all‐cause mortality, the association was directionally consistent but did not reach statistical significance (adjusted HR 2.245, 95% CI 0.696–7.242; *p* = 0.176), likely reflecting limited statistical power due to the small number of deaths in the validation cohort (Table S5).

### 3.5 Visualization of PII Predictive Efficacy and Comparison With Other Inflammatory Markers

**FIGURE 5:**
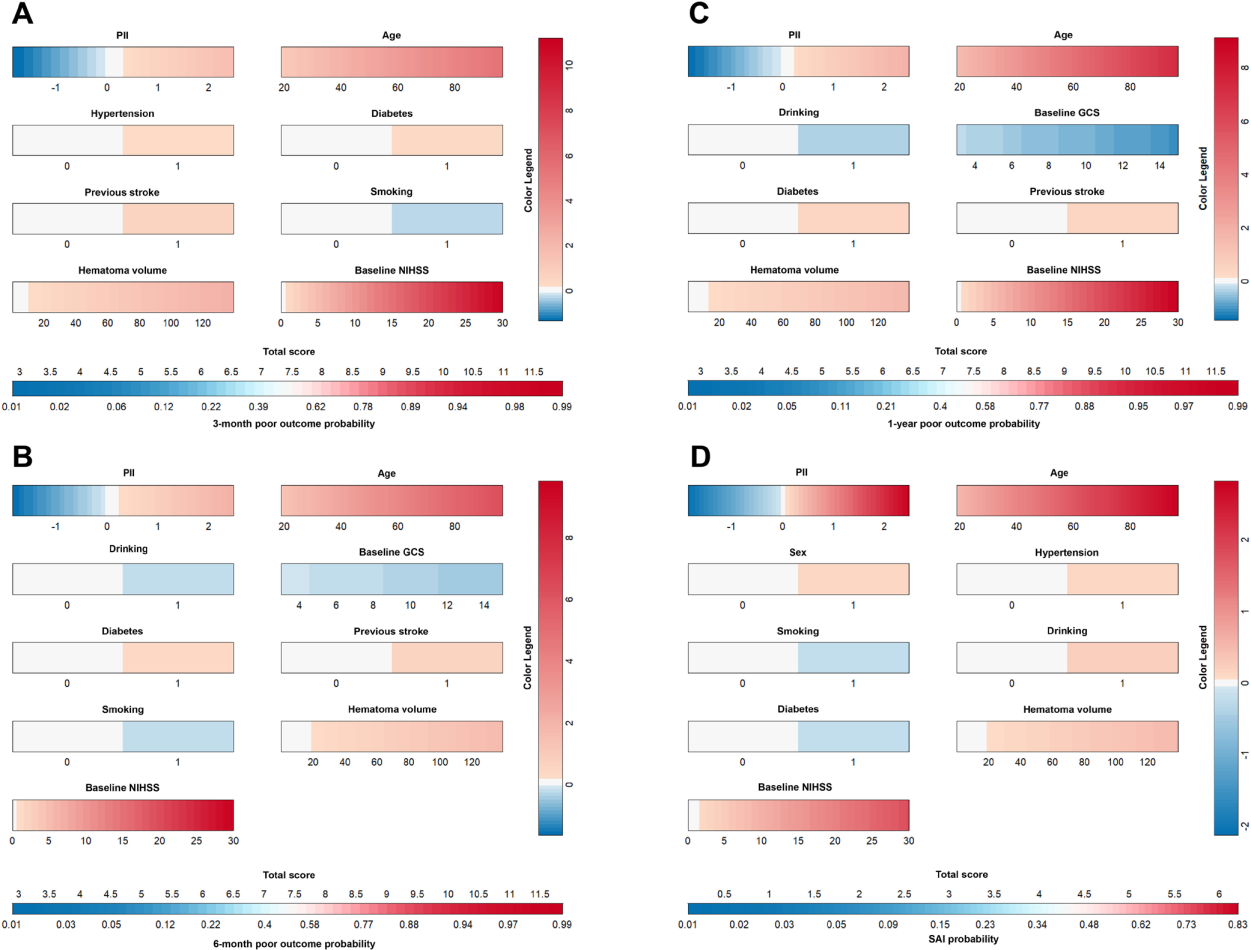
Nomogram illustrating the predictive performance of PII for poor outcomes at 3, 6, and 12 months, and SAI in ICH patients. (A) The relationship between PII and 3‐month poor outcomes in patients. (B) The relationship between PII and 6‐month poor outcomes in patients. (C) The relationship between PII and 1‐year poor outcomes in patients. (D) The relationship between PII and SAI in patients. GCS, Glasgow Coma Scale; ICH, intracerebral hemorrhage; NIHSS, National Institutes of Health Stroke Scale; PII, prognostic inflammation index; SAI, stroke‐associated infection.

To demonstrate the predictive efficacy of PII for poor outcomes, a nomogram was constructed (Figure 5). In the nomogram, red indicates higher scores and a greater likelihood of poor outcomes, while blue indicates lower scores and a reduced risk. Higher PII values were represented by deeper red shades, indicating stronger correlations with poor outcomes. PII demonstrated high predictive importance in the model. The C‐index for the 3‐month poor outcome model was 0.906, for the 6‐month model 0.903, for the 1‐year model 0.901, and for the SAI model 0.769.

We further compared PII with common systemic inflammation markers, including the systemic immune‐inflammation index (SII), systemic inflammation response index (SIRI), and aggregate inflammation systemic index (AISI) (Figure S3). The results showed that PII outperformed SII, SIRI, and AISI in predicting poor prognosis at 3 months (*p* < 0.001, *p* = 0.040, and *p* < 0.001, respectively). Additionally, PII demonstrated superior predictive power over SII and AISI for poor prognosis at 6 months (*p* < 0.001 and *p* < 0.001, respectively) and at 1 year (*p* < 0.001 and *p* < 0.001, respectively). Furthermore, PII was more effective than SII in predicting 1‐year all‐cause mortality (*p* = 0.013). Because these are multiple pairwise AUC comparisons, the reported *p* values are nominal; therefore, borderline comparisons should be interpreted cautiously and warrant external validation.

### 3.6 Longitudinal Changes in PII and Outcome Prediction

**FIGURE 6:**
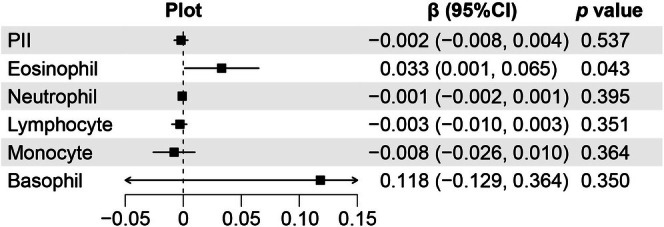
Longitudinal changes in prognosis prediction for ICH patients based on PII and its components. ICH, intracerebral hemorrhage; PII, prognostic inflammation index.

To evaluate whether PII and its components could predict longitudinal changes in outcomes, a generalized linear mixed‐effects model was used (Figure 6). Among PII components (eosinophil count, neutrophil count, monocyte count, lymphocyte count, and basophil count), only eosinophil count showed statistical significance in predicting 1‐year longitudinal outcome deterioration (β = 0.033, *p* = 0.043), while the other components did not reach statistical significance.

### 3.7 Sensitivity Analysis

To address potential confounding by secondary etiologies, we repeated the analyses after excluding all patients with secondary ICH (e.g., AVM/aneurysm, Moyamoya disease, and CAA). The results were consistent with the primary findings: higher PII remained significantly associated with poor functional outcomes at 3 months (adjusted OR 2.073, 95% CI 1.401–3.067; *p* < 0.001), 6 months (adjusted OR 2.410, 95% CI 1.618–3.588; *p* < 0.001), and 1 year (adjusted OR 2.197, 95% CI 1.475–3.272; *p* < 0.001), as well as with stroke‐associated infection (adjusted OR 3.235, 95% CI 2.185–4.788; *p* < 0.001) and 1‐year all‐cause mortality (adjusted HR 2.220, 95% CI 1.362–3.618; *p* = 0.001) (Table S1).

To account for potential coagulation‐related confounding, we additionally adjusted the fully adjusted models for platelet count. The results were materially unchanged: higher PII remained associated with poor functional outcome at 3 months (adjusted OR 2.056, 95% CI 1.385–3.054; *p* < 0.001), 6 months (adjusted OR 2.391, 95% CI 1.598–3.576; *p* < 0.001), and 1 year (adjusted OR 2.206, 95% CI 1.475–3.301; *p* < 0.001), stroke‐associated infection (adjusted OR 3.259, 95% CI 2.198–4.831; *p* < 0.001), and 1‐year all‐cause mortality (adjusted HR 2.131, 95% CI 1.282–3.542; *p* = 0.004) (Table S2).

We also refined the analysis of stroke‐associated infection by examining infection subtypes and accounting for baseline pulmonary susceptibility. In models additionally adjusted for pre‐existing pulmonary disease, higher PII was significantly associated with pulmonary infection (adjusted OR 3.160, 95% CI 2.086–4.786; *p* < 0.001), urinary tract infection (adjusted OR 3.349, 95% CI 1.173–9.567; *p* = 0.024), and other infections (adjusted OR 3.908, 95% CI 1.387–11.010; *p* = 0.010) (Table S3).

Given the potential influence of intraventricular hemorrhage on prognosis, we further performed a sensitivity analysis with additional adjustment for the presence of intraventricular hemorrhage. Higher PII remained significantly associated with poor functional outcome at 3 months (adjusted OR 1.804, 95% CI 1.211–2.687; *p* = 0.004), 6 months (adjusted OR 2.109, 95% CI 1.408–3.159; *p* < 0.001), and 1 year (adjusted OR 1.916, 95% CI 1.279–2.869; *p* = 0.002), stroke‐associated infection (adjusted OR 2.910, 95% CI 1.955–4.332; *p* < 0.001), and 1‐year all‐cause mortality (adjusted HR 2.036, 95% CI 1.243–3.335; *p* = 0.005) (Table S4).

## 4 Discussion

This study developed a novel PII to predict poor outcomes in patients with ICH. Our findings demonstrated that higher PII values were significantly associated with poor outcomes at 3, 6, and 12 months, as well as with SAI and higher 1‐year all‐cause mortality. Furthermore, higher PII trajectory groups were linked to an increased risk of these adverse outcomes. The nomogram showed that the PII prediction model exhibited excellent predictive performance and generally favorable discrimination compared with commonly used systemic inflammatory markers across most outcomes.

Inflammation plays a pivotal role in secondary brain injury induced by ICH. The inflammatory response is further exacerbated by various stimuli following ICH onset. Hematoma components activate microglia, initiating inflammatory signaling pathways and releasing pro‐inflammatory cytokines and chemokines, which attract peripheral inflammatory infiltration [18]. Concurrently, pyroptosis‐related pores formed after ICH release inflammatory cytokines, such as interleukin (IL)‐1β and IL‐18, which intensify the local inflammatory response, resulting in neuronal death, blood–brain barrier (BBB) disruption, and other types of secondary inflammatory injuries in ICH [19, 20].

Leukocyte subsets captured by the PII may represent an integrated systemic readout of the staged neuroimmune response after ICH. Following hematoma formation, activated microglia release pro‐inflammatory cytokines and chemokines that rapidly recruit peripheral immune cells into the injured brain tissue [4, 21]. Neutrophils are among the earliest infiltrating cells and can aggravate secondary brain injury by releasing pro‐inflammatory mediators, inducing oxidative stress, and disrupting the blood–brain barrier, thereby promoting cerebral edema and neurological deterioration [22, 23]. In parallel, monocytes are mobilized from the bone marrow into the circulation during the acute phase of ICH; while a subset may exert immunoregulatory and neuroprotective effects, sustained pro‐inflammatory stimulation can override these protective responses and is associated with unfavorable functional recovery [24]. Consistent with this pathophysiological stage, neutrophils and monocytes received positive weights in our RRR‐derived pattern, suggesting that the positive component of the PII predominantly reflects an early‐dominant and detrimental innate immune activation that is tightly linked to poor prognosis.

Conversely, the negative‐weighted components of the PII may reflect the suppression/exhaustion of protective immune pathways. Lymphocytes contribute to limiting secondary injury by restraining excessive microglial activation and preserving blood–brain barrier homeostasis [25]; however, the severe inflammatory cascade after ICH frequently triggers stroke‐induced immunodepression, characterized by impaired lymphocyte proliferation and lymphopenia, which increases susceptibility to secondary infection and worsens neurological outcomes [26, 27]. Eosinophils participate in thrombotic homeostasis and anti‐inflammatory tissue repair, and their depletion is a common immunological signature in acute critical vascular events associated with poor outcomes [28]. Basophils can modulate inflammatory remodeling through anti‐inflammatory cytokines (e.g., IL‐4 and IL‐13) [29], yet their quantity and function may also be compromised under systemic immune suppression. In our cohort, lymphocytes, eosinophils, and basophils were reduced and showed negative weights in the RRR model, collectively indicating an exhausted anti‐inflammatory/reparative pathway in patients at higher risk of adverse outcomes.

Importantly, the specific weighting pattern identified by RRR provides a biologically interpretable “imbalance axis” after ICH: predominance of deleterious innate immune responses (positive weights) accompanied by suppression and exhaustion of protective anti‐inflammatory and tissue‐repairing immunity (negative weights). This integrated pro‐inflammatory/anti‐inflammatory disequilibrium, captured in a single composite index, may explain why the PII showed superior prognostic performance compared with conventional ratio‐based inflammatory markers.

This study has several strengths. First, we developed a novel inflammation index and demonstrated its superiority over traditional systemic inflammatory markers in predicting poor outcomes in ICH patients, highlighting its potential clinical value. Second, PII is easily accessible, as it can be calculated using leukocyte counts from routine blood tests. However, this study also has certain limitations. It is a single‐center study with a relatively small sample size, which may introduce selection bias and reduce accuracy. Although we performed a temporal internal validation in an independent, non‐overlapping 2024 cohort, multicenter external validation remains necessary to further establish the generalizability of the PII. In the internal validation cohort, the number of deaths was limited, which may have reduced statistical power for mortality analyses and resulted in wide confidence intervals; therefore, the mortality finding should be interpreted cautiously. We evaluated multiple outcomes and conducted multiple pairwise AUC comparisons; we did not apply a formal correction for multiple testing, and therefore borderline ROC comparison *p* values should be interpreted as nominal and require confirmation in independent cohorts. Additionally, the study did not include other inflammatory factors such as IL‐6, which require additional testing and are less accessible than PII. Key coagulation‐related variables (e.g., INR) and detailed antithrombotic exposure (anticoagulant/antiplatelet use) were not available in a complete and standardized manner for all participants, which precluded uniform adjustment in multivariable models and may have introduced residual confounding. Additionally, baseline hematoma volume was estimated using the ABC/2 method, which is widely used in routine clinical practice but may be less accurate for irregular hematoma morphologies, and planimetric/segmentation‐based validation was not feasible in this retrospective dataset.

## 5 Conclusions

This study developed a novel RRR‐derived prognostic inflammation index (PII) incorporating five leukocyte subsets in ICH patients. PII and its trajectories were associated with poor outcomes and related endpoints. PII showed superior predictive performance compared with commonly used systemic inflammatory markers, supporting its potential utility for prognostic assessment in ICH.

## Author Contributions

D.Y., Y.W. conceptualized this work. D.Y., Y.W. supervised the study. G.C., F.C., X.L., Z.Z., F.C., Q.L., Z.R., C.Z., Y.Z., Y.C. and S.H. acquired the data, and G.C., F.C. and X.L. performed the statistical analysis and interpreted the data. G.C., F.C. and X.L. prepared the manuscript. D.Y., Y.W., G.C., F.C., X.L., Z.Z., F.C., Q.L., Z.R., C.Z., Y.Z., Y.C. and S.H. revised the manuscript. All authors approved the protocol.

## Funding

This work was supported by the National Innovation and Entrepreneurship Training Program for College Students (No. 202410343059), Zhejiang Provincial Science and Technology Innovation Program (New Young Talent Program) for College Students (No. 2025R413A039), and Wenzhou Municipal Sci‐Tech Bureau Program (No. Y20210585).

## Ethics Statement

This study was approved by the Ethics Committee of the First Affiliated Hospital of Wenzhou Medical University (Project No. KY2023‐R123) and was conducted in accordance with the Declaration of Helsinki. Given the retrospective design of the study, the requirement for informed consent for the analysis of existing data was waived by the Ethics Committee in accordance with national legislation and institutional requirements.

## Consent

The authors have nothing to report.

## Conflicts of Interest

The authors declare no conflicts of interest.

## Supporting information


**Table S1:** Sensitivity analysis of the associations between PII and clinical outcomes after excluding secondary intracerebral hemorrhage.
**Table S2:** Sensitivity analysis of the associations between PII and clinical outcomes with additional adjustment for platelet count.
**Table S3:** Multivariable associations between PII and specific types of stroke associated infection, with additional adjustment for preexisting pulmonary disease.
**Table S4:** Sensitivity analysis of the associations between PII and clinical outcomes with additional adjustment for the presence of intraventricular hemorrhage.
**Table S5:** Multivariable associations between PII and clinical outcomes in the internal validation cohort.
**Table S6:** Summary of missing data in the derivation and validation cohorts.
**Table S7:** Bootstrap internal validation of RRR‐derived loadings for PII construction.
**Figure S1:** The flowchart showing the patient selection process.
**Figure S2:** Classification of ICH patients based on the dynamic trajectory of PII.
**Figure S3:** Comparison of the predictive performance of PII and common systemic inflammatory markers for poor prognosis in patients.
**Figure S4:** RRR factor structure and leukocyte loading pattern used to derive the Prognostic Inflammation Index.
(A) Average variation explained in the response variables by the first two reduced rank regression (RRR) factors. Factor 1 explained substantially more variation than Factor 2 (14.7408% vs. 0.0141%), supporting the use of the dominant factor for constructing the PII.
(B) Leukocyte subset loadings for the selected RRR factor used to define the PII. Positive loadings were observed for neutrophils (0.393) and monocytes (0.057), whereas negative loadings were observed for lymphocytes (−0.223), eosinophils (−0.057), and basophils (−0.045). Orange bars indicate positive loadings and blue bars indicate negative loadings.

## Data Availability

The data that support the findings of this study are available from the corresponding author upon reasonable request.

## References

[1] “Global, Regional, and National Burden of Stroke and Its Risk Factors, 1990–2019: A Systematic Analysis for the Global Burden of Disease Study 2019,” Lancet Neurology 20 (2021): 795–820. 34487721 10.1016/S1474-4422(21)00252-0 PMC8443449

[2] L. Xu , Z. Wang , W. Wu , M. Li , and Q. Li , “Global, Regional, and National Burden of Intracerebral Hemorrhage and Its Attributable Risk Factors From 1990 to 2021: Results From the 2021 Global Burden of Disease Study,” BMC Public Health 24 (2024): 2426. 39243077 10.1186/s12889-024-19923-7 PMC11378620

[3] D. Woo , M. E. Comeau , S. U. Venema , et al., “Risk Factors Associated With Mortality and Neurologic Disability After Intracerebral Hemorrhage in a Racially and Ethnically Diverse Cohort,” JAMA Network Open 5 (2022): e221103. 35289861 10.1001/jamanetworkopen.2022.1103 PMC8924717

[4] R. F. Keep , Y. Hua , and G. Xi , “Intracerebral Haemorrhage: Mechanisms of Injury and Therapeutic Targets,” Lancet Neurology 11 (2012): 720–731. 22698888 10.1016/S1474-4422(12)70104-7 PMC3884550

[5] Z. Hu , S. Chen , E. Zhang , et al., “Novel Inflammatory Markers in Intracerebral Hemorrhage: Results From Olink Proteomics Analysis,” FASEB Journal 39 (2025): e70341. 39853806 10.1096/fj.202402183RR PMC11760662

[6] T. Wang , D. Nowrangi , L. Yu , et al., “Activation of Dopamine D1 Receptor Decreased NLRP3‐Mediated Inflammation in Intracerebral Hemorrhage Mice,” Journal of Neuroinflammation 15 (2018): 2. 29301581 10.1186/s12974-017-1039-7 PMC5753458

[7] Y. R. Kim , Y. M. Kim , J. Lee , J. Park , J. E. Lee , and Y. M. Hyun , “Neutrophils Return to Bloodstream Through the Brain Blood Vessel After Crosstalk With Microglia During LPS‐Induced Neuroinflammation,” Frontiers in Cell and Development Biology 8 (2020): 613733. 10.3389/fcell.2020.613733 PMC7753044 33364241

[8] S. Mei , Y. Shao , Y. Fang , et al., “The Changes of Leukocytes in Brain and Blood After Intracerebral Hemorrhage,” Frontiers in Immunology 12 (2021): 617163. 33659003 10.3389/fimmu.2021.617163 PMC7917117

[9] G. Zhao , Y. Gu , Z. Wang , Y. Chen , and X. Xia , “The Clinical Value of Inflammation Index in Predicting ICU Mortality of Critically Ill Patients With Intracerebral Hemorrhage,” Frontiers in Public Health 12 (2024): 1373585. 39157528 10.3389/fpubh.2024.1373585 PMC11327062

[10] P. Guo and W. Zou , “Neutrophil‐To‐Lymphocyte Ratio, White Blood Cell, and C‐Reactive Protein Predicts Poor Outcome and Increased Mortality in Intracerebral Hemorrhage Patients: A Meta‐Analysis,” Frontiers in Neurology 14 (2023): 1288377. 38288330 10.3389/fneur.2023.1288377 PMC10824245

[11] R. H. Wang , W. X. Wen , Z. P. Jiang , et al., “The Clinical Value of Neutrophil‐To‐Lymphocyte Ratio (NLR), Systemic Immune‐Inflammation Index (SII), Platelet‐to‐Lymphocyte Ratio (PLR) and Systemic Inflammation Response Index (SIRI) for Predicting the Occurrence and Severity of Pneumonia in Patients With Intracerebral Hemorrhage,” Frontiers in Immunology 14 (2023): 1115031. 36860868 10.3389/fimmu.2023.1115031 PMC9969881

[12] K. Guldolf , F. Vandervorst , R. Gens , A. Ourtani , T. Scheinok , and S. De Raedt , “Neutrophil‐To‐Lymphocyte Ratio Predicts Delirium After Stroke,” Age and Ageing 50 (2021): 1626–1632. 34218276 10.1093/ageing/afab133

[13] A. Morotti , J. Nawabi , A. Pilotto , et al., “Functional Outcome Improvement From 3 to 12 Months After Intracerebral Hemorrhage,” European Stroke Journal 9 (2024): 391–397. 38183279 10.1177/23969873231222782 PMC11318429

[14] R. U. Kothari , T. Brott , J. P. Broderick , et al., “The ABCs of Measuring Intracerebral Hemorrhage Volumes,” Stroke 27 (1996): 1304–1305. 8711791 10.1161/01.str.27.8.1304

[15] S. Suda , J. Aoki , T. Shimoyama , et al., “Stroke‐Associated Infection Independently Predicts 3‐Month Poor Functional Outcome and Mortality,” Journal of Neurology 265 (2018): 370–375. 29249057 10.1007/s00415-017-8714-6

[16] Y. Wang , Y. Wang , R. Li , et al., “Low‐Grade Systemic Inflammation Links Heavy Metal Exposures to Mortality: A Multi‐Metal Inflammatory Index Approach,” Science of the Total Environment 947 (2024): 174537. 38977088 10.1016/j.scitotenv.2024.174537

[17] J. Zhao , F. Yuan , C. Song , et al., “Safety and Efficacy of Three Enteral Feeding Strategies in Patients With Severe Stroke in China (OPENS): A Multicentre, Prospective, Randomised, Open‐Label, Blinded‐Endpoint Trial,” Lancet Neurology 21 (2022): 319–328. 35219379 10.1016/S1474-4422(22)00010-2

[18] Y. Zhou , Y. Wang , J. Wang , R. Anne Stetler , and Q. W. Yang , “Inflammation in Intracerebral Hemorrhage: From Mechanisms to Clinical Translation,” Progress in Neurobiology 115 (2014): 25–44. 24291544 10.1016/j.pneurobio.2013.11.003

[19] H. Zhu , Z. Wang , J. Yu , et al., “Role and Mechanisms of Cytokines in the Secondary Brain Injury After Intracerebral Hemorrhage,” Progress in Neurobiology 178 (2019): 101610. 30923023 10.1016/j.pneurobio.2019.03.003

[20] D. Song , C. T. Yeh , J. Wang , and F. Guo , “Perspectives on the Mechanism of Pyroptosis After Intracerebral Hemorrhage,” Frontiers in Immunology 13 (2022): 989503. 36131917 10.3389/fimmu.2022.989503 PMC9484305

[21] M. Xue and V. W. Yong , “Neuroinflammation in Intracerebral Haemorrhage: Immunotherapies With Potential for Translation,” Lancet Neurology 19 (2020): 1023–1032. 33212054 10.1016/S1474-4422(20)30364-1

[22] L. Puy , A. R. Parry‐Jones , E. C. Sandset , D. Dowlatshahi , W. Ziai , and C. Cordonnier , “Intracerebral Haemorrhage,” Nature Reviews. Disease Primers 9 (2023): 14. 10.1038/s41572-023-00424-7 36928219

[23] K. Shi , D. C. Tian , Z. G. Li , A. F. Ducruet , M. T. Lawton , and F. D. Shi , “Global Brain Inflammation in Stroke,” Lancet Neurology 18 (2019): 1058–1066. 31296369 10.1016/S1474-4422(19)30078-X

[24] S. X. Shi , K. Shi , and Q. Liu , “Brain Injury Instructs Bone Marrow Cellular Lineage Destination to Reduce Neuroinflammation,” Science Translational Medicine 13 (2021): 13. 10.1126/scitranslmed.abc7029 33853930

[25] L. L. Mao , H. Yuan , W. W. Wang , et al., “Adoptive Regulatory T‐Cell Therapy Attenuates Perihematomal Inflammation in a Mouse Model of Experimental Intracerebral Hemorrhage,” Cellular and Molecular Neurobiology 37 (2017): 919–929. 27678140 10.1007/s10571-016-0429-1 PMC11482213

[26] U. Dirnagl , J. Klehmet , J. S. Braun , et al., “Stroke‐Induced Immunodepression: Experimental Evidence and Clinical Relevance,” Stroke 38 (2007): 770–773. 17261736 10.1161/01.STR.0000251441.89665.bc

[27] A. Morotti , S. Marini , M. J. Jessel , et al., “Lymphopenia, Infectious Complications, and Outcome in Spontaneous Intracerebral Hemorrhage,” Neurocritical Care 26 (2017): 160–166. 28004330 10.1007/s12028-016-0367-2 PMC5336513

[28] Q. Chen , J. Liu , H. Xu , et al., “Association Between Eosinophilic Leukocyte Count and Hematoma Expansion in Acute Spontaneous Intracerebral Hemorrhage,” Frontiers in Neurology 10 (2019): 1164. 31736868 10.3389/fneur.2019.01164 PMC6834787

[29] F. Sicklinger , I. S. Meyer , X. Li , et al., “Basophils Balance Healing After Myocardial Infarction via IL‐4/IL‐13,” Journal of Clinical Investigation 131 (2021): e136778, 10.1172/JCI136778. 34196299 PMC8245180

