# Supplementary material for: Development of a Novel Prognostic Inflammation Index to Predict Poor Outcomes in Patients With Intracerebral Hemorrhage: A Longitudinal Study
Source: CNS Neurosci Ther. 2026 Apr 27;32(4):e70904. doi: 10.1002/cns.70904 (PMC13121913; doi:10.1002/cns.70904)
Supplement: Supplementary file 1 — Table S1: Sensitivity analysis of the associations between PII and clinical outcomes after excluding secondary intracerebral hemorrhage. Table S2: Sensitivity analysis of the associations between PII and clinical outcomes with additional adjustment for platelet count. Table S3: Multivariable associations between PII and specific types of stroke associated infection, with additional adjustment for preexisting pulmonary disease. Table S4: Sensitivity analysis of the associations between PII and clinical outcomes with additional adjustment for the presence of intraventricular hemorrhage. Table S5: Multivariable associations between PII and clinical outcomes in the internal validation cohort. Table S6: Summary of missing data in the derivation and validation cohorts. Table S7: Bootstrap internal validation of RRR‐derived loadings for PII construction. Figure S1: The flowchart showing the patient selection process. Figure S2: Classification of ICH patients based on the dynamic trajectory of PII. Figure S3: Comparison of the predictive performance of PII and common systemic inflammatory markers for poor prognosis in patients. Figure S4: RRR factor structure and leukocyte loading pattern used to derive the Prognostic Inflammation Index. (A) Average variation explained in the response variables by the first two reduced rank regression (RRR) factors. Factor 1 explained substantially more variation than Factor 2 (14.7408% vs. 0.0141%), supporting the use of the dominant factor for constructing the PII. (B) Leukocyte subset loadings for the selected RRR factor used to define the PII. Positive loadings were observed for neutrophils (0.393) and monocytes (0.057), whereas negative loadings were observed for lymphocytes (−0.223), eosinophils (−0.057), and basophils (−0.045). Orange bars indicate positive loadings and blue bars indicate negative loadings. [file CNS-32-e70904-s001.docx]

**SUPPLEMENTARY MATERIALS**

**Development of a Novel Prognostic Inflammation Index to Predict Poor Outcomes in Patients with Intracerebral Hemorrhage: A Longitudinal Study**

**
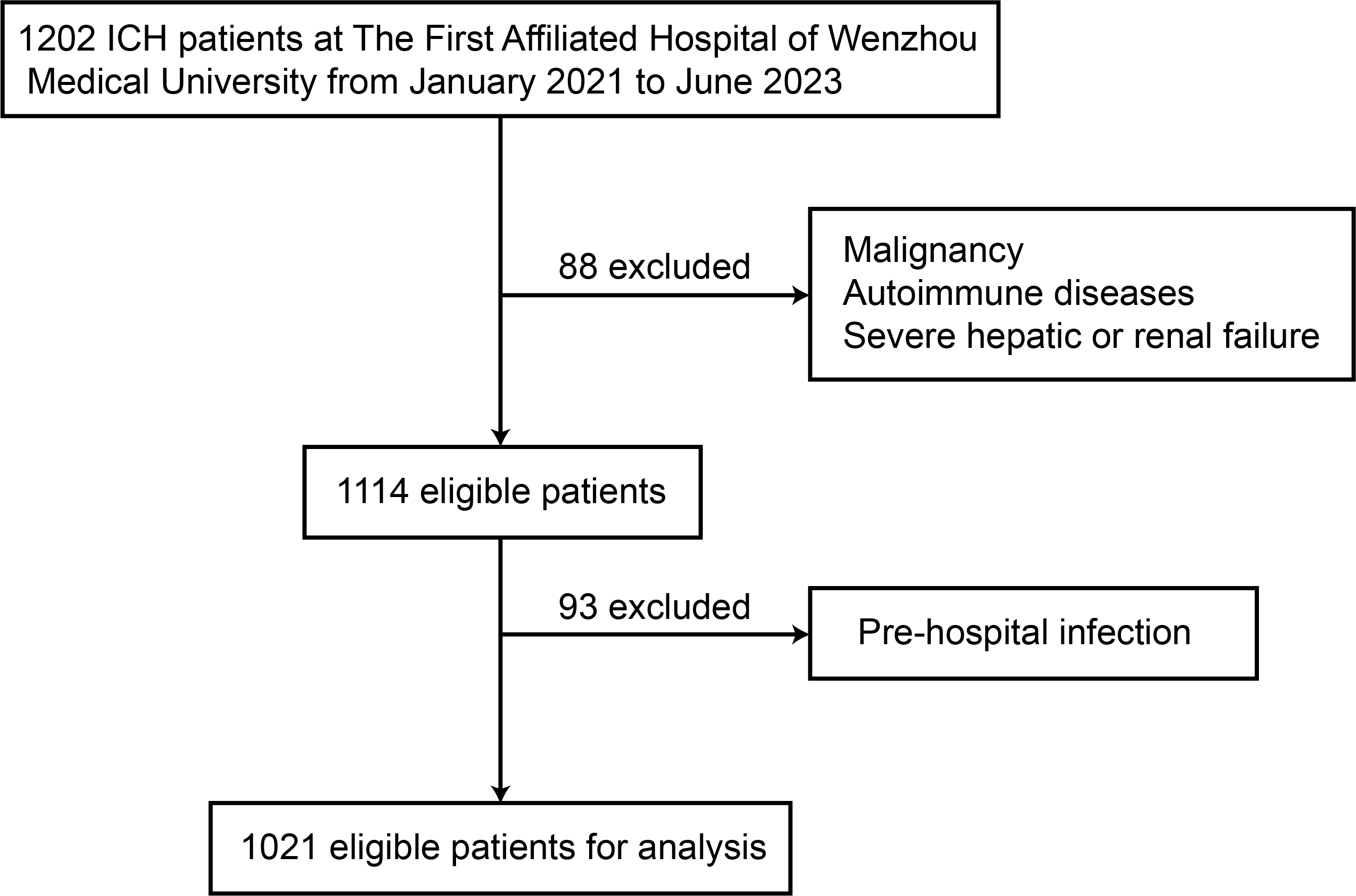
**

**Supplementary Figure 1.** The flowchart showing the patient selection process.


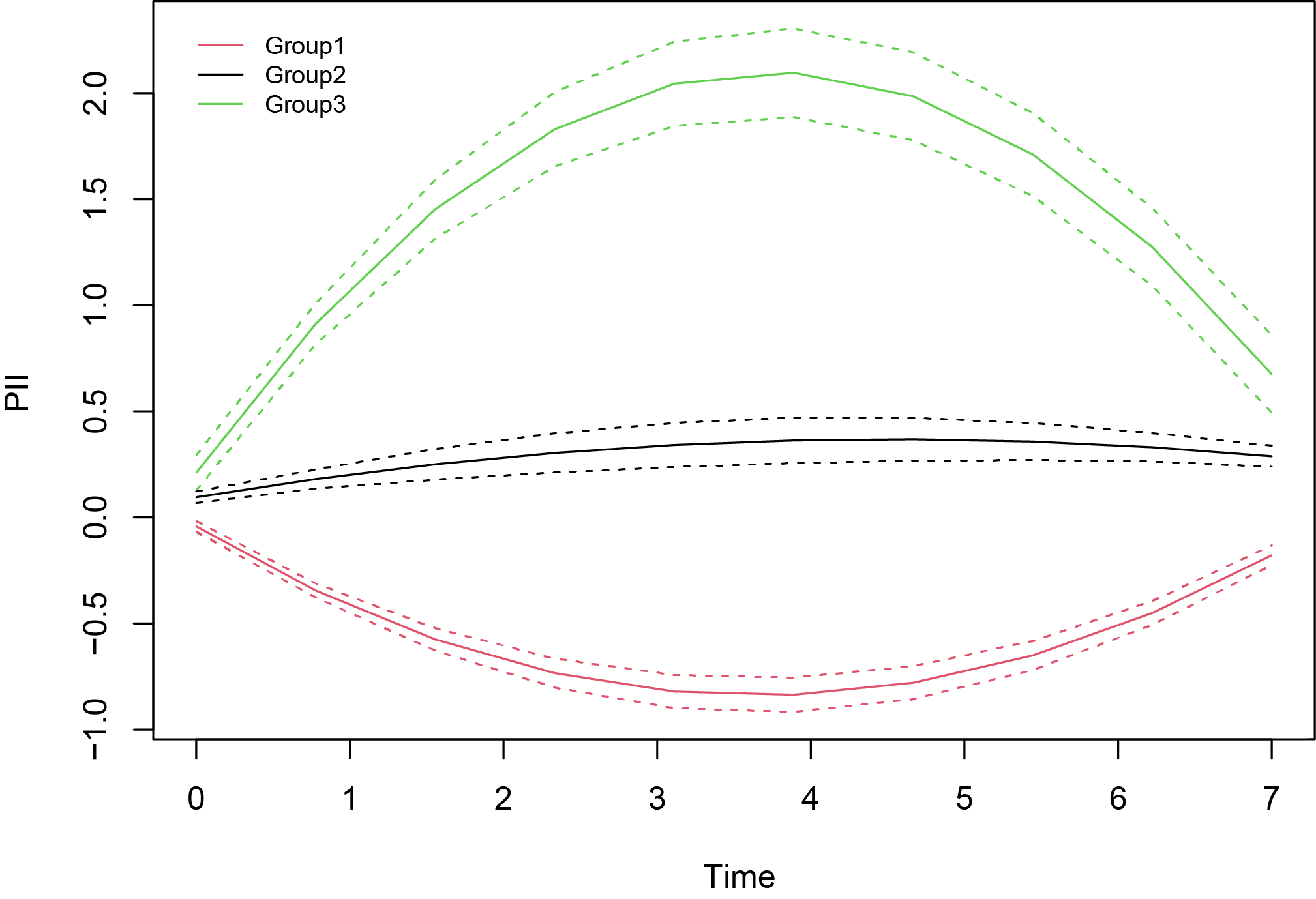


**Supplementary Figure 2.** Classification of ICH patients based on the dynamic trajectory of PII.

**Abbreviations:** PII, prognostic inflammation index; ICH, intracerebral hemorrhage.


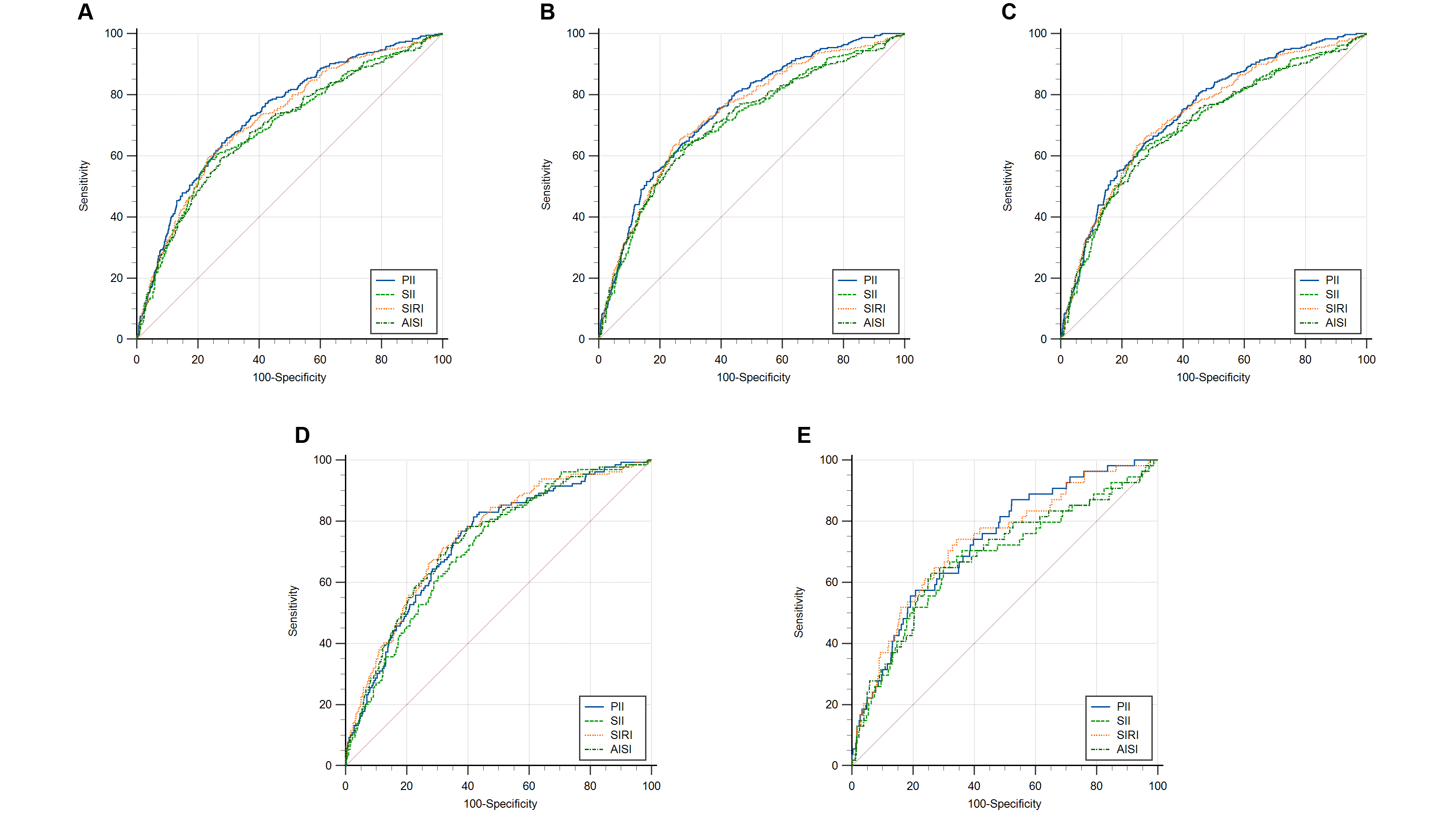


**Supplementary Figure 3.** Comparison of the predictive performance of PII and common systemic inflammatory markers for poor prognosis in patients

**Abbreviations:** PII, prognostic inflammation index; SII, systemic immune-inflammation index; SIRI, systemic inflammation response index; AISI, aggregate inflammation systemic index.


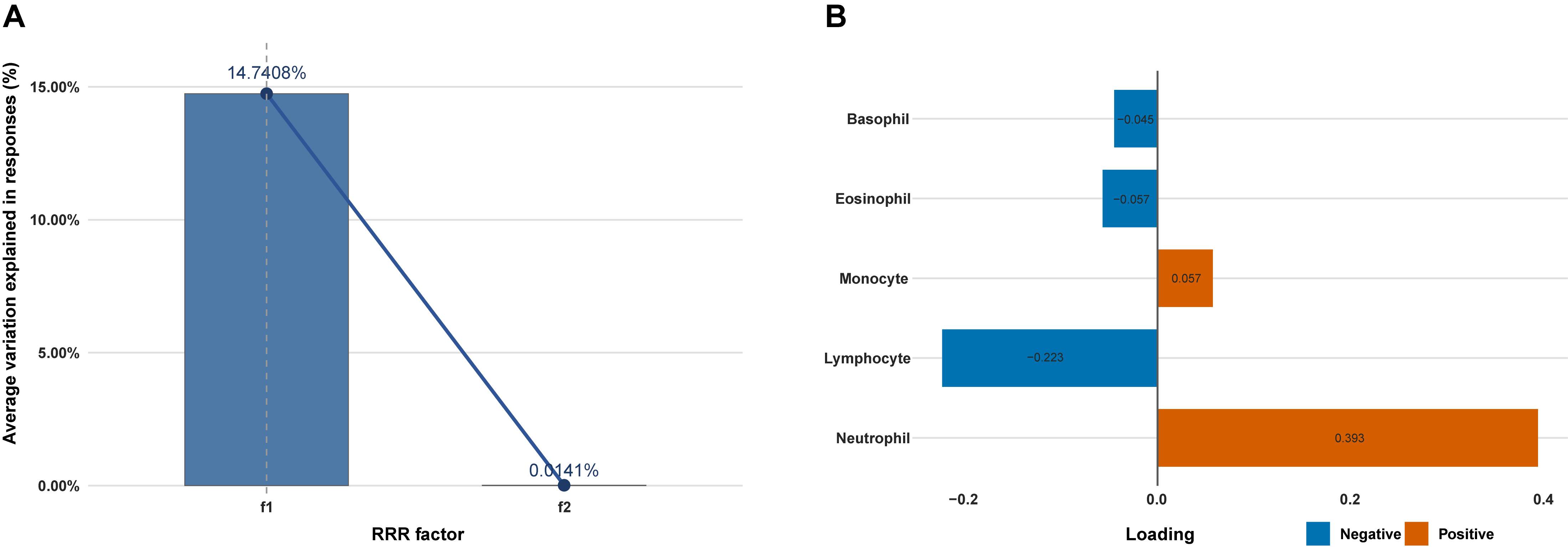


**Supplementary Figure 4.** RRR factor structure and leukocyte loading pattern used to derive the Prognostic Inflammation Index.

**(A)** Average variation explained in the response variables by the first two reduced rank regression (RRR) factors. Factor 1 explained substantially more variation than Factor 2 (14.7408% vs 0.0141%), supporting the use of the dominant factor for constructing the PII. **(B)** Leukocyte subset loadings for the selected RRR factor used to define the PII. Positive loadings were observed for neutrophils (0.393) and monocytes (0.057), whereas negative loadings were observed for lymphocytes (−0.223), eosinophils (−0.057), and basophils (−0.045). Orange bars indicate positive loadings and blue bars indicate negative loadings.

**Abbreviations:** RRR, reduced rank regression; PII, prognostic inflammation index.

**Supplementary Table1.** Sensitivity analysis of the associations between PII and clinical outcomes after excluding secondary intracerebral hemorrhage.

| **Outcomes** |  | | **PII** |
| --- | --- | --- | --- |
| 3-month poor outcomes | Model 1 | OR ( 95% CI ) | 5.192 (3.824, 7.049) |
|  |  | *p*-value | < 0.001 |
|  | Model 2 | OR ( 95% CI ) | 2.073 (1.401, 3.067) |
|  |  | *p*-value | < 0.001 |
| 6-month poor outcomes | Model 1 | OR ( 95% CI ) | 5.837 (4.230, 8.054) |
|  |  | *p*-value | < 0.001 |
|  | Model 2 | OR ( 95% CI ) | 2.410 (1.618, 3.588) |
|  |  | *p*-value | < 0.001 |
| 1-year poor outcomes | Model 1 | OR ( 95% CI ) | 5.604 (4.049, 7.775) |
|  |  | *p*-value | < 0.001 |
|  | Model 2 | OR ( 95% CI ) | 2.197 (1.475, 3.272) |
|  |  | *p*-value | < 0.001 |
| SAI | Model 1 | OR ( 95% CI ) | 3.740 (2.653, 5.274) |
|  |  | *p*-value | < 0.001 |
|  | Model 2 | OR ( 95% CI ) | 3.235 (2.185, 4.788) |
|  |  | *p*-value | < 0.001 |
| 1-year all-cause mortality | Model 1 | HR ( 95% CI ) | 3.305 (2.073, 4.443) |
|  |  | *p*-value | < 0.001 |
|  | Model 2 | HR ( 95% CI ) | 2.220 (1.362, 3.618) |
|  |  | *p*-value | 0.001 |

**Abbreviations:** SAI, stroke-associated infection; PII, prognostic inflammation index; ICH, intracerebral hemorrhage, OR, odds ratio; HR, hazard ratio; CI, confidence interval.

**Model 1** adjusted for age and sex.

**Model 2** adjusted for age, sex, smoking, drinking, hypertension, diabetes, previous stroke, hematoma volume, baseline NIHSS, baseline GCS.

**Supplementary Table 2.** Sensitivity analysis of the associations between PII and clinical outcomes with additional adjustment for platelet count.

| **Outcomes** | **PII** | |
| --- | --- | --- |
| 3-month poor outcomes | OR ( 95% CI ) | 2.056 (1.385, 3.054) |
|  | *p*-value | < 0.001 |
| 6-month poor outcomes | OR ( 95% CI ) | 2.391 (1.598, 3.576) |
|  | *p*-value | < 0.001 |
| 1-year poor outcomes | OR ( 95% CI ) | 2.206 (1.475, 3.301) |
|  | *p*-value | < 0.001 |
| SAI | OR ( 95% CI ) | 3.259 (2.198, 4.831) |
|  | *p*-value | < 0.001 |
| 1-year all-cause mortality | HR ( 95% CI ) | 2.131 (1.282, 3.542) |
|  | *p*-value | 0.004 |

**Abbreviations:** SAI, stroke-associated infection; PII, prognostic inflammation index; PLT, platelet count; ICH, intracerebral hemorrhage, OR, odds ratio; HR, hazard ratio; CI, confidence interval.

**Model:** adjusted for age, sex, smoking, drinking, hypertension, diabetes, previous stroke, hematoma volume, baseline NIHSS, baseline GCS, PLT.

**Supplementary Table 3.** Multivariable associations between PII and specific types of stroke associated infection, with additional adjustment for preexisting pulmonary disease.

| **Outcomes** | **PII** | |
| --- | --- | --- |
| Pulmonary infection | OR ( 95% CI ) | 3.160 (2.086, 4.786) |
|  | *p*-value | < 0.001 |
| Urinary tract infection | OR ( 95% CI ) | 3.349 (1.173, 9.567) |
|  | *p*-value | 0.024 |
| Other infections | OR ( 95% CI ) | 3.908 (1.387, 11.010) |
|  | *p*-value | 0.010 |

**Abbreviations:** SAI, stroke-associated infection; PII, prognostic inflammation index; ICH, intracerebral hemorrhage, OR, odds ratio; HR, hazard ratio; CI, confidence interval.

**Model:** adjusted for age, sex, smoking, drinking, hypertension, diabetes, previous stroke, hematoma volume, baseline NIHSS, baseline GCS, preexisting pulmonary disease.

**Supplementary Table 4.** Sensitivity analysis of the associations between PII and clinical outcomes with additional adjustment for the presence of intraventricular hemorrhage.

| **Outcomes** | **PII** | |
| --- | --- | --- |
| 3-month poor outcomes | OR ( 95% CI ) | 1.804 (1.211, 2.687) |
|  | *p*-value | 0.004 |
| 6-month poor outcomes | OR ( 95% CI ) | 2.109 (1.408, 3.159) |
|  | *p*-value | < 0.001 |
| 1-year poor outcomes | OR ( 95% CI ) | 1.916 (1.279, 2.869) |
|  | *p*-value | 0.002 |
| SAI | OR ( 95% CI ) | 2.910 (1.955, 4.332) |
|  | *p*-value | < 0.001 |
| 1-year all-cause mortality | HR ( 95% CI ) | 2.036 (1.243, 3.335) |
|  | *p*-value | 0.005 |

**Abbreviations:** SAI, stroke-associated infection; PII, prognostic inflammation index; ICH, intracerebral hemorrhage, OR, odds ratio; HR, hazard ratio; CI, confidence interval.

**Model:** adjusted for age, sex, smoking, drinking, hypertension, diabetes, previous stroke, hematoma volume, baseline NIHSS, baseline GCS, intraventricular hemorrhage.

**Supplementary Table 5.** Multivariable associations between PII and clinical outcomes in the internal validation cohort.

| **Outcomes** | **PII** | |
| --- | --- | --- |
| 3-month poor outcomes | OR ( 95% CI ) | 2.285 (1.171, 4.458) |
|  | *p*-value | 0.015 |
| 6-month poor outcomes | OR ( 95% CI ) | 3.175 (1.522, 6.622) |
|  | *p*-value | 0.002 |
| 1-year poor outcomes | OR ( 95% CI ) | 2.897 (1.410, 5.952) |
|  | *p*-value | 0.004 |
| SAI | OR ( 95% CI ) | 3.342 (1.787, 6.248) |
|  | *p*-value | < 0.001 |
| 1-year all-cause mortality | HR ( 95% CI ) | 2.245 (0.696, 7.242) |
|  | *p*-value | 0.176 |

**Abbreviations:** SAI, stroke-associated infection; PII, prognostic inflammation index; ICH, intracerebral hemorrhage, OR, odds ratio; HR, hazard ratio; CI, confidence interval.

**Note:** The number of deaths in the internal validation cohort was small; therefore, the mortality estimate may be unstable and should be interpreted with caution.

**Model:** adjusted for age, sex, smoking, drinking, hypertension, diabetes, previous stroke, hematoma volume, baseline NIHSS, baseline GCS.

**Supplementary Table 6.** Summary of missing data in the derivation and validation cohorts.

| Characteristics | Frequency of missing data | |
| --- | --- | --- |
|  | Derivation cohort | Validation cohort |
| Missing 24-hour CBC leukocyte-related inflammatory parameters | 59 | 66 |
| Lack of complete hematoma volume | 38 | 35 |
| Lack of baseline NIHSS and GCS assessments | 2 | 47 |
| Missing data on covariates | 4 | 0 |
| Lack of complete follow-up mRS assessments at 3, 6, and 12 months | 5 | 0 |

**Supplementary Table 7.** Bootstrap internal validation of RRR-derived loadings for PII construction.

| **Predictor** | **Loading** | **2.5%** | **97.5%** | **Sign consistency** |
| --- | --- | --- | --- | --- |
| Eosinophil | -0.056789 | -0.134068 | 0.017932 | 93.6% |
| Neutrophil | 0.393489 | 0.279857 | 0.417644 | 100.0% |
| Monocyte | 0.057043 | -0.037652 | 0.165633 | 87.4% |
| Lymphocyte | -0.222789 | -0.286900 | -0.124367 | 100.0% |
| Basophil | -0.044896 | -0.121634 | 0.026281 | 87.0% |

**Legend:** Loadings correspond to the coefficients of the first reduced-rank regression (RRR) latent factor used to construct the Prognostic Inflammation Index (PII) in the derivation cohort. Predictors (leukocyte subset counts) were standardized before RRR fitting. The 2.5th and 97.5th percentiles represent the bootstrap-based 95% confidence interval obtained from 1,000 resamples with sign alignment across resamples. Sign consistency indicates the proportion of bootstrap resamples in which the loading retained the same sign as in the original RRR model.
